# Genetic structure and population diversity of eleven edible herbs of Eastern Crete

**DOI:** 10.1186/s40709-015-0030-7

**Published:** 2015-05-30

**Authors:** Antonia Psaroudaki, Nikolaos Nikoloudakis, Georgios Skaracis, Andreas Katsiotis

**Affiliations:** Department of Crop Science, Laboratory of Plant Breeding and Biometry, Agricultural University of Athens, 75 Iera Odos, Athens, Greece; Department of Nutrition and Dietetics, Technological Educational Institute of Crete, Crete, Tripitos, Sitia Greece; Department of Agricultural Science, Biotechnology and Food Science, Cyprus University of Technology, Athinon and Anexartisias 57, 3603 Limassol, Cyprus

**Keywords:** Compositae, Umbelliferae, Labiatae, Primulaceae, DNA amplification fingerprinting, AMOVA

## Abstract

**Background:**

The present work aimed to investigate the genetic structure of 11 edible herbs grown in the wild of eastern Crete that are becoming vulnerable due to habitat destruction and unregulated harvesting. Thirty three populations (268 individuals) of *Reichardia picroides*, *Scolymus hispanicus*, *Scandix pecten-veneris*, *Leontodon tuberosus*, *Cichorium spinosum, Sonchus asper* ssp*. glaucescens*, *Urospermum picroides*, *Prasium majus*, *Hypochoeris radicata*, *Centaurea raphanina* ssp*. raphanina* and *Anagallis arvensis* were collected and identified from nine regions with distinct microclimate (Lassithi prefecture), and their genetic composition was studied by means of RAPD markers.

**Results:**

A total of ten primers per population were used to detect genetic diversity and bootstrap analysis was conducted for clustering the samples. High levels of heterogeneity were revealed while the Analysis of Molecular Variance documented that variance was allocated mainly within populations and at a lesser extent among populations. *Fst* values among regions were moderate to high, suggesting partial population fragmentation. Bayesian structure analysis revealed fine genetic composition and substantial admixture between species present in different regions, although clustering was mainly geographically related.

**Conclusions:**

High altitude regions, with little residential and agricultural development (Kefala, Agrilos, Ziros and Tziritis), were the areas where high biodiversity was detected. On the other hand, coastal regions had lower biodiversity, probably due to degradation of their habitat.

## Background

Greece is a country where more than six thousand plant species are listed, from which almost 500 of them are indigenous. At the island of Crete alone about 2000 different taxa have been recorded [1]. Local endemism is a rather common feature of the island, which shelters more than 140 indigenous species [1]. In addition, low plant species homogeneity exists among different regions of Crete. This has been attributed to the natural geographical barriers, such as high mountains, and the different microclimates between the island’s regions [1]. Due to its geographical location, Lassithi in particular presents a unique flora, being in the eastern part of Crete and at the most southern part of Europe.

Another distinct feature of the Cretan vegetation is that many herbs, endemic or not, are edible and constitute an integral part of the everyday traditional diet. Several studies on wild edible herbs consumed in Crete even today have demonstrated their great nutritional value [2–6]. However, up to now there have not been any studies concerning the biodiversity of wild edible species populations in eastern Crete. This is essential for any program relating to the conservation and exploitation of genetic resources (*in situ* and/or *ex situ*) that are under threat, mainly because of over-harvesting from the wild (it is difficult to cultivate them and no proper guidelines exist) as well as by the agricultural intensification and urbanization. Recently, a directive from the Greek forestry department was issued for all regions in Crete prohibiting collecting from the wild of edible plants for marketing reasons, while restricted quantities are allowed for personal use, in order to promote the growth of natural populations.

To acquire a general overview of the genetic diversity of 11 edible wild species, 33 populations were collected from nine different locations of eastern Crete (all located in the Lassithi region) totaling 268 individuals. Surveying morphological variation of wild species is a rather cumbersome and difficult task since little (if any) morphological descriptors have been developed and there is always the chance that collectors may remove specimens included in any genetic study. In the present study plants were collected for botanical identification as well as for DNA fingerprinting using RAPD markers. We aimed to address: a) the extent of genetic diversity of the sampled populations, b) the within and between population genetic diversity, and c) the genetic structure of species/populations related to their distribution. The ultimate purpose is to identify areas where species exhibit greater variability for *ex situ* collection, *in situ* preservation and possibly propagation of genetic material.

## Results

### Genetic diversity

**Fig. 1:**
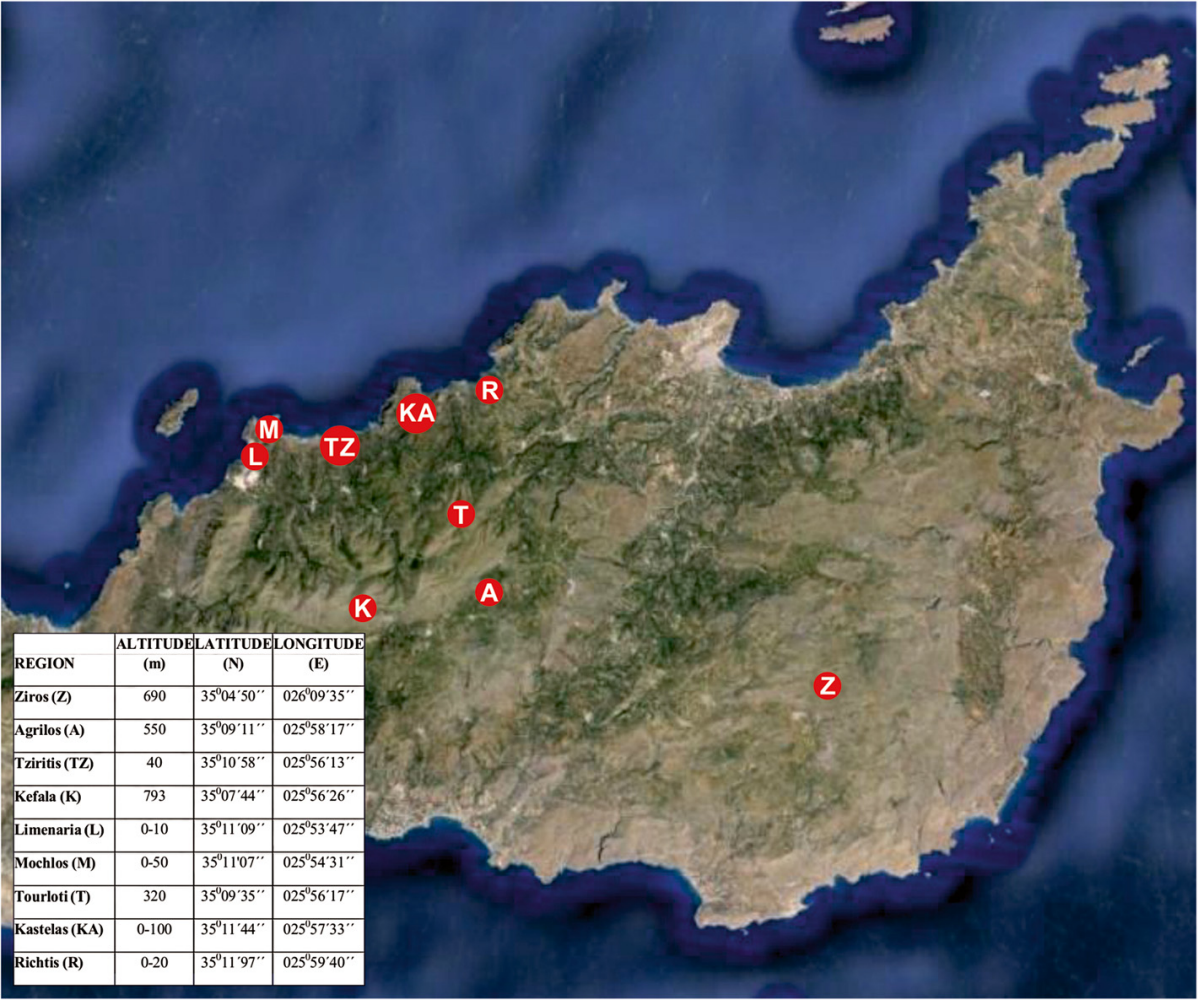
Location and coordinates of the eastern Crete sampled edible weed populations

In total, 268 plants were identified and studied in their natural habitat (Figure 1; Table 1). Selected primers (Table 2) provided sufficient polymorphism (more than 80%) in most cases. Specifically, one primer alone (OPAH-16) produced polymorphic fragments for eight of the eleven species (100% for *S. hispanicus*, 100% for *L. tuberosus*, 91.66% for *C. raphanina* ssp*. raphanina,* 90.90% for *A. arvensis*, 88.88% for *R. picroides,* 70% for *U. picroides*, 66.66% for *H. radicata* and 60% for *P. majus*)*.* Mean heterozygosity (Table 3) was higher in *S. hispanicus* (0.271 ± 0.017), *C. raphanina* Sm*.* ssp*. raphanina* (0.253 ± 0.013) and *H. radicata* (0.246 ± 0.016), while the lowest values were recorded for *A. arvensis* (0.182 ± 0.012), *S. asper* subsp*. glaucescens* (0.181 ± 0.009) and *P. majus* (0.140 ± 0.008).

**Table 1:** Number of taxa collected, identified and analyzed

	*Reichardia picroides*	*Scolymus hispanicus*	*Scandix pecten – veneris*	*Leontodon tuberosus*	*Cichorium spinosum*	*Urospermum picroides*	*Prasium majus*	*Hypochoeris radicata*	*Centaurea raphanina* ssp*. raphanina*	*Anagallis arvensis*	*Sonchus asper* ssp*. glaucescens*	*Taxa collected*
Region												
Ziros	8	11	10	5	-	10	7	10	13	4	5	83
Αgrilos	10	-	12	-	-	12	-	12	-	6	7	59
Τziritis	8	-	4	10	-	9	11	-	-	3	5	50
Κefala	-	11	-	5	-	-	-	-	7	-	-	23
Limenaria	-	-	-	5	-	-	2	-	-	-	-	7
Mochlos	-	-	-	-	-	-		-	-	-	2	2
Τοurloti	-	-	15	-	-	-	9	-	-	-	-	24
Κastelas	-	-	-	-	10	-	-	-	-	-	-	10
Richtis	-	-	-	-	10	-	-	-	-	-	-	10
Taxa collected	26	22	41	25	20	31	29	22	20	13	19	268

**Table 2:** Species studied and primers used

Vernacular name and systematics	Primers used
ΑGALATSIDA	OPAH-16, OPB-8, OPA-7, OPG-5,
*Reichardia picroides* (L. Roth)	OPAI-12, OPAI-14, OPN-8, OPN-19,
Compositae	OPAI-5, OPAH-9
ASKOLYMPROS	OPAH-2, OPAH-16, OPB-8, OPAH-
*Scolymus hispanicus*	18, OPAI-5, OPAI-8, OPAI-11, OPA-
Compositae	7, OPAI-14
ACHARTZIKAS	OPAI-5, OPAI-14, OPAI-8, OPAI-12,
*Scandix pecten-veneris* (L)	OPA-7, OPA-20, OPAH-2, OPAH-11,
Umbelliferae	OPG-5, OPAH-1
VIZORADIKO	OPAH-1, OPAH-2, OPAH-9, OPAI-
*Leontodon tuberosus*	11, OPAH-11, OPAI-14, OPA-7, OPG-
Compositae	5, OPA-20, OPAH-16
GIALORADIKO	OPA-20, OPAH-1, OPAH-9, OPAH-
*Cichorium spinosum*	18, OPAI-11, OPA-7, OPO-8, OPG-5,
Compositae	OPB-8, OPM-19
ZOCHOS	OPA-7, OPAI-5, OPAI-12, OPAI-13,
*Sonchus asper* ssp*. glaucescens*	OPAH-9, OPAH-18, OPAI-8, OPAI-
Compositae	14, OPA-7, OPO-8
KORKOLEKANIDA	OPAH-1, OPAH-2, OPAH-9, OPAH-
*Urospermum picroides*	11, OPAH-16, OPAH-17, OPAH-18,
Compositae	OPAI-5, OPAI-11, OPA-20
LAGOUTO	OPG-5, OPB-8, OPB-1, OPA-7,
*Prasium majus* (L)	OPAH-1, OPAH-2, OPAH-11, OPAH-
Labiatae	16, OPAH-17, OPAH-18
PACHIES	OPAH-1, OPAH-2, OPAH-9, OPAH-
*Hypochoeris radicata*	16, OPAH-17, OPAI-5, OPB-1, OPB-2
Compositae	
PETROKARA	OPAI-12, OPAH-16, OPB-5, OPB-8,
*Centaurea raphanina* ssp*. raphanina*	OPB-11, OPB-13, OPG-5, OPI-1,
Compositae	OPM-19
POLYNTERI	OPAH-1, OPAH-2, OPAH-9, OPAH-
*Anagallis arvensis*	16, OPAH-17, OPAH-18, OPAI-8,
Primulaceae	OPAI-12, OPAI-14

**Table 3:** Mean heterogeneity (*He*) and standard error for the species studied

	*Reichardia picroides*	*Scolymus hispanicus*	*Scandix pecten – veneris*	*Leontodon tuberosus*	*Cichorium spinosum*	*Urospermum picroides*	*Prasium majus*	*Hypochoeris radicata*	*Centaurea raphanina* ssp*. raphanina*	*Anagallis arvensis*	*Sonchus asper* ssp*. glaucescens*
**Region**											
Ziros	0.238 ± 0.021	0.258 ± 0.024	0.218 ± 0.021	0.213 ± 0.020	-	0.205 ± 0.021	0.161 ± 0.018	0.172 ± 0.022	0.247 ± 0.018	**0.213 ± 0.021**	0.192 ± 0.018
Αgrilos	0.215 ± 0.021	-	0.185 ± 0.022	-	-	0.238 ± 0.021	-	**0.319 ± 0.020**	-	0.144 ± 0.020	**0.236 ± 0.016**
Τziritis	**0.249 ± 0.020**	-	0.197 ± 0.022	0.199 ± 0.020	-	**0.256 ± 0.023**	0.158 ± 0.019	-	-	0.190 ± 0.019	0.176 ± 0.019
Κefala	-	**0.283 ± 0.024**	-	**0.275 ± 0.017**	-	-	-	-	0.259 ± 0.018	-	-
Limenaria	-	-	-	0.183 ± 0.020	-	-	0.075 ± 0.015	-	-	-	-
Mochlos	-	-	-	-	-	-		-	-	-	0.118 ± 0.017
Τοurloti	-	-	**0.242 ± 0.022**	-	-	-	**0.166 ± 0.018**	-	-	-	-
Κastelas	-	-	-	-	0.234 ± 0.020	-	-	-	-	-	-
Richtis	-	-	-	-	**0.297 ± 0.020**	-	-	-	-	-	-
**Average** ***He***	0.234 ± 0.012	0.271 ± 0.017	0.211 ± 0.011	0.218 ± 0.10	0.238 ± 0.012	0.233 ± 0.012	0.140 ± 0.008	0.246 ± 0.016	0.253 ± 0.013	0.182 ± 0.012	0.181 ± 0.009

Maximum values per species are emphasized

High levels of genetic heterogeneity (Table 3) were detected, while the Analysis of Molecular Variance (AMOVA) (Table 4) partitioned the genetic variance mainly within populations (*S. hispanicus,* 95%; *U. picroides,* 87%; *R. picroides,* 87%; *C. spinosum,* 85%). On the other hand, the lowest within population diversity was recorded for *S. asper* ssp*. glaucescens* (69%) that also had the highest *Fst* values (0.310) revealing high levels of divergence.

**Table 4:** Analysis of Molecular Variance (AMOVA) for the species studied

Variation	*Reichardia picroides*	*Scolymus hispanicus*	*Scandix pecten – veneris*	*Leontodon tuberosus*	*Cichorium spinosum*	*Urospermum picroides*	*Prasium majus*	*Hypochoeris radicata*	*Centaurea raphanina* ssp*. raphanina*	*Anagallis arvensis*	*Sonchus asper* ssp*. glaucescens*
Among pop^a^	13%	5%	22%	18%	15%	13%	15%	20%	16%	-	31%
Within pop^a^	87%	95%	78%	82%	85%	87%	85%	80%	84%	-	69%
*Fst* (*p* > 0.001)	0.134	0.050	0.220	0.180	0.154	0.129	0.148	0.197	0.157	-	0.310

^a^Only populations with more than five individuals were considered

**Fig. 2:**
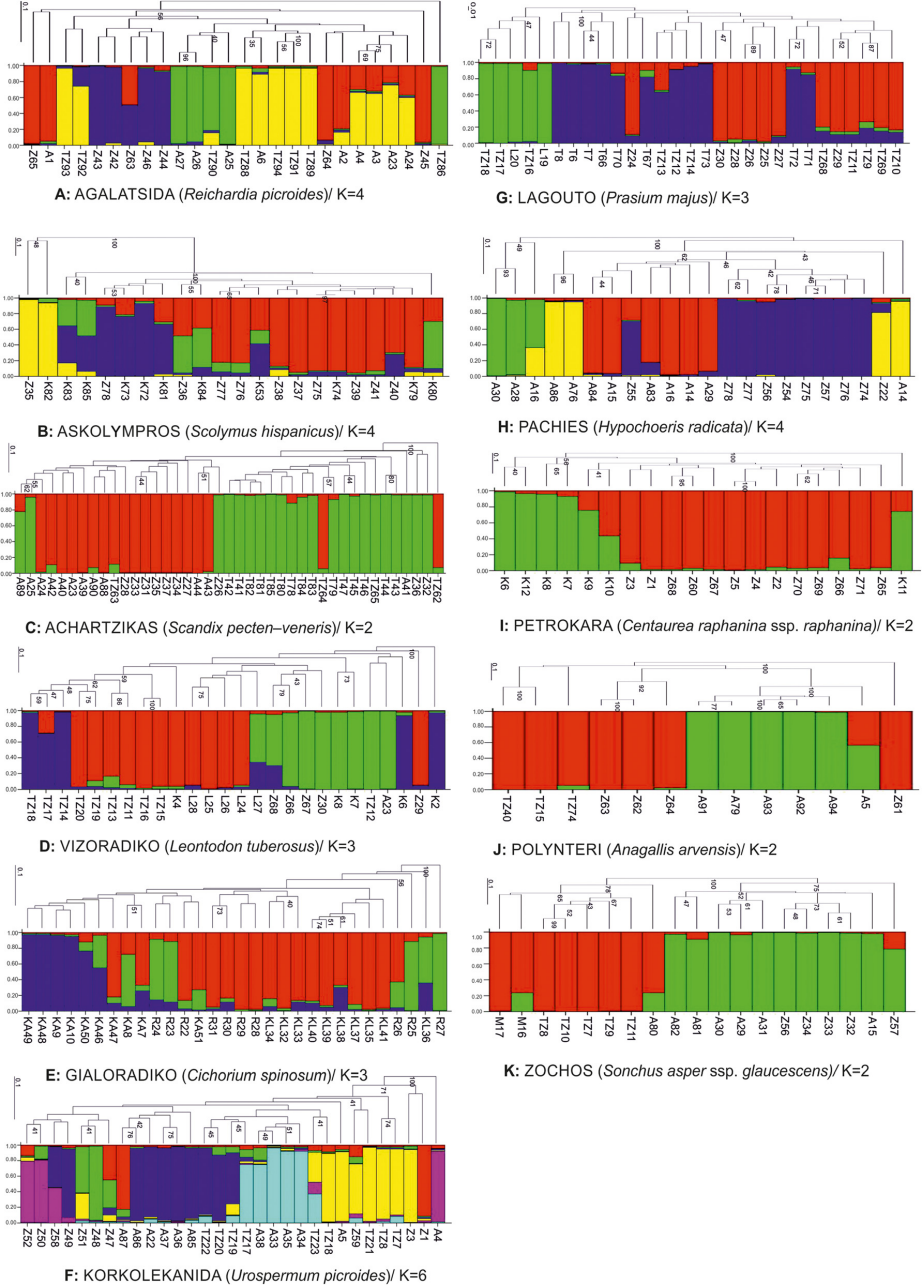
Bootstrap analysis and Bayesian cluster analysis of the optimum K cluster, for each species studied. Bootstrap values greater than 40 % are shown. The colour in each bar plot represents the probability of each individual belonging to a given group. **a**: Αgalatsida; **b**: Askolympros; **c**: Achartzikas; **d**: Vizoradiko; **e**: Gialoradiko; **f**: Korkolekanida; **g**: Lagouto; **h**: Pachies; **i**: Petrokara; **j**: Polynteri; **k**: Zochos

In general, samples were organized in small clusters and further divided in subgroups, showing moderate and high bootstrap values (Figure 2). In several instances, individuals from different populations tended to group together. In the cases of *S. pecten-veneris*, *C. spinosum*, *H. radicata*, *C. raphanina* spp*. raphanina*, *A. arvensis* and *S. asper* spp*. glaucescens* a definite region-oriented clustering was recorded. Overall, extensive admixture was recorded among accessions of different populations and several subclusters were formed. On the contrary, other populations seemed to cluster together due to reduced genetic diversity among them. Results for each species are as follows:

#### *Reichardia picroides* (agalatsida)

High affinity was recorded among individuals from Τziritis and Αgrilos, while accessions from Ziros revealed a unique genetic composition, although extensive admixture was recorded, as illustrated in Figure 2a. The lowest genetic similarity occurred between populations of Tziritis and Ziros, which are the most geographically distant areas, while the Τziritis population was the most heterogeneous (Table 3). Moderate *Fst* values were recorded among populations and variation occurred almost exclusively within populations (Table 4).

#### *Scolymus hispanicus* (askolymbros)

Samples were clustered according to their geographic origin. The region with the highest diversity was Kefala (Table 3) and extensive admixture was recorded among populations. A population-oriented clustering was not detected by neither the dendrogram nor the Bayesian analysis. Surprisingly, two distinctive accessions (ZC358 and K1E827) that were of unique genetic structure, were grouped together and diverged from the rest (Figure 2b). AMOVA partitioned the genetic diversity almost exclusively within populations (95%) while differentiation among populations was minute (*Fst* = 0.05; Table 4).

#### *Scandix pecten-veneris* (achartzikas)

The highest population diversity was detected in the Tourloti region (Table 3). Accessions belonging to the neighboring regions of Tziritis and Tourloti were highly affiliated, as recorded by both the dendrogram and the Bayesian analysis (Figure 2c). Also, individuals from Agrilοs were more related to those from Ziros. Twenty two percent of the overall genetic variation was attributed among populations and *Fst* values suggested substantial divergence (Table 4).

#### *Leontodon tuberosus* (vizoradiko)

The highest diversity within a population was detected at Kefala (Table 3). Accessions from Tziritis were clustered together and supported by high bootstrap values, although the Bayesian analysis showed that there were mainly three genetic subgroups with minimum genetic admixture (Figure 2d). High affinity was recorded among the accessions of Limenaria and Ziros regions, but a number of individuals remained ungrouped. In addition, relatively high within population variability and moderate *Fst* values according to AMOVA were recorded (Table 4).

#### *Cichorium spinosum* (gialoradiko)

A definite geographic clustering was observed for this species. Accessions from Κastelas and cultivated *C. spinosum* formed distinctive clusters, having nevertheless fair affinity to the Richtis group, which was the most variable (Table 3) and genetically admixtured (Figure 2e). According to the AMOVA, the variability among populations reached 15% and fair *Fst* values were obtained (Table 4).

#### *Urospermum picroides* (korkolekanida)

This species had the most complex organization since individuals from all regions formed small affiliated subclusters (Figure 2). The most diverged populations were from Τziritis and Αgrilos (Table 3). However individuals from the Ziros region formed two highly admixtured connecting clusters that encompass elements from the whole *U. picroides* gene pool (Figure 2f). Also, the Αgrilos population was subdivided revealing high similarity to the Τziritis individuals. As a result, genetic variance was allocated within populations, although *Fst* values suggested moderate deviation (Table 4).

#### *Prasium majus* (lagouto)

Individuals were grouped mainly according to their region of origin forming subclusters (Figure 2g). The main diversity occurs within the Τοurloti and Ziros (Table 3) populations and minor admixture was detected among populations. The diversity was allocated mainly within populations (85%) while moderate variance was recorded among populations (Table 4).

#### *Hypochoeris radicata* (pahies)

The most variable population was located in Αgrilos (Table 3). In addition, Argilos individuals were further subdivided, with each subcluster showing a unique genetic composition (Figure 2h). In general, plants were clustered according to their collection sites and especially those from Ziros were highly affiliated (indicated by bootstrap values) and had a distinctive genetic profile. The only exception was one accession that remained unrelated and was placed in the Αgrilos subcluster (Figure 2). Genetic variation for *H. radicata* was attributed mainly within populations (80%) as indicated by AMOVA and populations were fairly diverged (*Fst* = 0.197; Table 4).

### *Centaurea raphanina* Sm. subsp. *raphanina (petrokara)*

The Kefala region is the area where the highest biodiversity of *C. raphanina* Sm*.* subsp*. raphanina* occurs, as indicated in Table 3. On the other hand, individuals from Ziros are tightly connected with little genetic admixture (Figure 2i). Little affinity could be the result of population alienation (*Fst* = 0.157). In this species it seems that variability mainly exists within populations (84%; Table 4).

#### *Anagallis arvensis* (polynteri)

Accessions were organized according to geographic origin and clustering was supported by high bootstrap values and minimum genetic admixture (Figure 2j). High affinity was observed among samples of the Tziritis and Ziros regions, while samples from Argilos were more distant. Both Ziros and Tziritis (Table 3) populations exhibited the highest amount of diversity.

### *Sonchus asper* subsp*. glaucescens (zochos)*

Accessions from the three regions formed distinct clusters with high bootstrap values, as illustrated in the dendrogram and almost no genetic admixture was detected by the Bayesian analysis (Figure 2k). The most variable population was from Αgrilos (Table 3). In addition, structure illustrated that accessions from Mochlos were genetically identical to the samples from Tziritis (coastal areas). Besides, high affinity was recorded among the accessions of Ziros and Αgrilos (upland areas), while substantial admixture of genotypes was not revealed (Figure 2). It should be noted that *S. asper* subsp. glaucescens was the species that had the highest among population variability and the highest *Fst* values in this study; hence admixture among populations was minimal.

## Discussion

Although scientific interest is increased for the Cretan diet, still very little information is available about the natural habitat, dispersal and genetic variability of Cretan edible annual and/or perennial plants. A few studies have focused on some of the genera included in the present study using molecular markers [7, 8], however samples from Greece were limited. It is generally accepted that information regarding the mode of reproduction (self-/cross-pollination), the life cycle (annual/perennial), the genetic structure and diversity of a plant species is essential for its conservation [9–11]. Moreover, considering that wild edible plants are potentially important food sources, it was necessary to study the genetic diversity of their natural gene pool and the spatial distribution in relation to eco-geographical factors.

For all species studied, genetic variability among populations was evident to a lesser or greater extent. For each species, the corresponding dendrogram showed a trend for population clustering according to geographic origin, which is consistent with other studies [12]. Furthermore, almost for all collection sites, clustering was found to be non-random, thus indicating a fine degree of genetic structure among the various populations (Figure 2). The present data suggest that the 11 studied species differed substantially in their genetic variability structure. Populations of the related Compositae *S. hispanicus* and *C. raphanina* ssp*. raphanina* had the highest average heterogeneity, followed by the moderately diverged *H. radicata* and *C. spinosum*. This is consistent with studies of natural populations, as genetic diversity among populations increases in relation to their geographical distance [12]. However, groups formed by individuals from selected regions, indicated that there is no isolation of populations, but in most areas there were entries related to another region, than to those of their own territory. That indicates intra-specific variability in zones that may be associated with cross-pollination [13]. Moreover, nearby populations were more genetically related, especially in the case of *S. pectenveneris*. It seems that cross-pollination and the lack of sequestered populations and remote regions has led to genetic homogenization among adjacent populations [13–16]. *Hypochoeris radicata* has been studied due to its recent and rather quick spread around the world. It is considered a species that easily colonizes diverse environments, preferring moist and cool places. In general, annual species with high germination percentage, fast growth and reproduction, show low within and high among population diversity [17]. Honnay *et al.* [18] and Ortiz *et al.* [8] refer to *H. radicata* as a self-pollinating, but primarily is considered as a cross-pollinating species. They proposed that the species in its ancestral hobs (North Africa) behaves as a cross-pollinator and that some individuals appear as self-pollinators. They can grow up quickly as self-pollinators in order to colonize an area and then behave as cross-pollinators resulting in a buildup of diversity [8]. In the region of Eastern Crete, the species occurs only in higher altitude areas (cooler), and presents moderate diversity within populations (possibly because there is a fair amount of self-pollination [19, 20]). This justifies the differentiation between areas where variability is relatively high.

Low genetic variability was found for *A. arvensis* and *S. asper* ssp*. glaucescens*, indicating population fragmentation that could lead to limited gene flow [21]. In isolated populations, insect pollinators cannot reach effortless distant populations and, therefore, populations tend to deteriorate [22–25]. Furthermore, the size of a population could relate positively to genetic diversity. This comes as no surprise for annual plants, since population size fluctuates year by year [26] and selfing species vary more than outbreeders regarding effective population size and levels of genetic diversity [13]. Moreover, small populations are prone to stochastic demographic events as well as genetic effects like inbreeding, genetic bottlenecks or even accumulation of deleterious mutations [27]. On the other hand, geographic isolation and undersized populations along with restricted gene flow frequently cause genetic drift and inbreeding [21, 28], leading to high genetic differentiation [29].

Heterozygosity varies greatly in other annual taxa examined e.g., He = 0.004 in *Cicer arietinum* [30], He = 0.005 to 0.049 for five different *Lens* taxa [31], or He = 0.241 in *Poa annua* [32]. This points to the fact that the mode of reproduction may not contribute considerably to gene diversity [33], although other studies suggest otherwise [17]. It is broadly accepted that the reproduction system influences gene flow dramatically [34–36]. Nybom & Bartish [33], have illustrated that by recording a mean of He around 0.09 for selfing species. In contrast, taxa with a mixed or outcrossing reproduction system have a He value of about 0.22 to 0.26. In the present study, the apparent division of individual plant populations according to geographic origin of collection, points out that the populations came from heterogeneous fragments and there were moderate levels of genetic differentiation among them within a region. In addition, the observed genetic similarity between individuals from different populations sometimes was greater compared to individuals from the same population (*R. picroides*, *L. tuberosus*). These observations show the dynamics of populations in various regions as well as the differentiation of areas concerning their biodiversity for all species of indigenous edible plants.

The data presented could facilitate a decision for the *in situ* conservation of genetic resources and the selection of protected areas. The regions of Kefala, Agrilos, Tziritis and Ziros presented important biodiversity for most of the species investigated, while the areas of Mochlos, Kastelas, Limenaria and Richitis had fewer species occurrences with reduced variability. The areas of Kefala, Agrilos, Tourloti and Ziros are located in relatively high altitude and have restricted residential development, therefore most species appear to have great heterogeneity. In the region of Kefala, the species *S. hispanicus, L. tuberosus,* and *C. raphanina* Sm. subsp*. raphanina* exhibit broad genetic diversity. In Agrilos great variability is documented for *S. asper* subsp*. glaucescens, U. picroides, H. radicata* and *A. arvensis,* while in Ziros for *R. picroides, S. pecten-veneris,* and *P. majus. Cichorium spinosum* was only found in coastal areas showing high diversity in the Richtis region. Finally at Tourloti, *S. pectin* and *P. majus* had the highest heterogeneity.

The above findings could be explained mainly on the basis of land use and the overall human activity in the regions. The areas with the lowest diversity include mainly cultivated land (olive trees and intensified vegetable cultivation) and large settlements with tourism development. The low levels of diversity in agricultural land could be caused by the pressure due to the cultivation techniques (plowing, weed control) and may be associated with an increase in inbreeding as the number of plants in a population decline since the only place where they reproduce is at the margins of cultivated land [15]. Furthermore, as arable plants are mainly short-lived and self-pollinated [37], genetic responses to increasing fragmentation and decreasing population size should be relatively rapid. It is generally acknowledged that modern agricultural practices have exposed autochthonous plant populations to serious survival pressure. Losses in arable plant communities have been larger than in most other human-made vegetation types, to the extent that arable plant communities now belong to the most threatened vegetation types [38].

In contrast, regions showing greater plant biodiversity are clearly less residential (Kefala and Agrilos have seasonal settlements and no permanent residence), with less intensified agriculture production (presence of perennial crops such as pome fruit and grapes, and ovine ranching). Furthermore, these areas are usually difficult to access, crossed by rugged rural dirt roads and only approached by shepherds and residents for seasonal works, with fallow land and limited ovine presence. Unfortunately, during the last years the Ziros plateau is becoming more accessible (paved roads) and more residential, while the grazing sheep/goat populations have increased due to internal migration from the west of Crete [39].

The species examined in the current study could provide an insight to the capacity of eastern Crete genetic resources for edible plants with potential economic value. The increased interest of consumers already has overcome the narrow base of the island, and has resulted in the commercial cultivation of some species (*C. spinosum* and *S. hispanicus*). This in turn has increased collection of edible plants from the wild for commercial purposes since collectors have a market with high retail prices. The unmonitored collection, which does not take place in the traditional way (i.e., few individuals per population for personal use), could lead to the decrease or even the extinction of a species.

The economy of collection has also shifted in other ways compared to the past, as older people could identify more plant species, thus collecting plants from several species to make their ‘daily dish of greens’. Younger people recognize fewer species, collect them with persistence and as a consequence disturb the planta equilibrium in a region [40].

Another major risk for the conservation of species and their diversity is the residential development, especially in recent years. Initially, this has affected coastal areas but now, in combination to tourism development, leads to the extinction of many indigenous plants, due to the loss of their habitat. Agricultural practices also have an effect in the areas where edible wild plants grow. For instance, at altitudes above 600 m extensive sowing of clover and other forage plants and deep plowing of soils occur; so there is more grass for small ruminants (sheep, goats), which in turn damages the biodiversity of the region.

The condition for the species examined is considered threatening. Furthermore, Ziros (located in the boundaries of the habitat CORINE Eastern Crete - A00050056, http://filotis.itia.ntua.gr/biotopes/c/A00050056/) is characterized as degraded/slow degrading, due to disturbances in the ecological balance caused by fires, tourism development and overgrazing, and must be prioritized as a preserved area. Kefala and Agrilos (similarly included in the habitat CORINE Sitia and Mount Ornos - A00040086, http://filotis.itia.ntua.gr/biotopes/c/A00040086/) are also designated as degraded/slow degrading. Given the fact that the recent land use changes and residential development apparently can affect the genetic structure of the Cretan edible plant communities, regions should be closely monitored over the coming years and develop conservation policies.

## Conclusions

In the present study it was recorded that the plant populations studied have a heterogeneous genetic composition and that substantial levels of genetic diversity exist within a region. This underlines the population dynamics in several Cretan areas along with the divergence of zones, regarding the biodiversity of indigenous edible plants of Lassithi. Furthermore, the regions of Kefala, Agrilos and Tziritis were identified as those where population heterogeneity peaked for most of the studied species. Urgent measures should be taken in order to protect these areas from habitat degradation.

## Methods

### Collection areas, species and sampling design

Global Positioning System (GPS) data of eastern Crete areas and plants of the present study (*Reichardia picroides, Scolymus hispanicus, Scandix pecten-veneris, Leontodon tuberosus, Cichorium spinosum, Sonchus asper* subsp*. glaucescens, Urospermum picroides, Prasium majus, Hypochoeris radicata, Centaurea raphanina* Sm*.* subsp*. raphanina* and *Anagallis arvensis*) are reported in Figure 1 and Table 1. Firstly, leaf samples were collected from labeled plants and were dried in silica gel at the vegetative stage (from November to February). Secondly, at the flowering season (from April to June) the same plants were collected for botanical taxonomy. Taxonomy was conducted by the Ecology & Classification Division of the Biology Department of the University of Athens and was based on full plant samples. For their classification the following literature was used: Flora Hellenica [41, 42], Flora Europaea [43, 44] and Flora of the Cretan Area [1]. The confirmation of sample identification was achieved through comparisons to botanical material that is stored at the Botanical Museum of the University of Athens (ΑΤΗU). Plant coding reflects the region of origin followed by the serial number of the sample collected. For instance, plant coded as Z42 (*Reichardia picroides*) was collected at Ziros and was the 42th sample acquired.

### DNA extraction and PCR reactions

DNA extraction was performed using the Nucleospin Plant II Kit (Macherey-Nagel) and the lysis buffer I, following manufacturer’s instructions. DNA concentration and quality was calculated spectrophotometrically (Unicam Helios; OD260nm/OD280nm ratios were above 1.8) and confirmed with 1% agarose electrophoresis using standard λ-phage molecular weights. More than 30 random decamer primers (Operon) were used for the initial screening of samples, from which nine or ten primers, depending on the species, were retained for their ability to produce unambiguous polymorphic products among genotypes (Table 2). PCR reactions were carried out in a 25 μL reaction mixture using 1X PCR reaction buffer (20 mM Tris–HCl pH 8.4, 50 mM KCl), 50 ng of the total DNA, 100 pmol of each decamer primer, 200 mM dNTP, 1 U Taq DNA polymerase (Promega) and 1.5 mM MgCl_2_. The cycling profile consisted of an initial denaturation step of 3 min, followed by 35 cycles of 30 s at 94°C, 45 s at 37°C, and 1 min at 72°C and a final elongation step of 10 min at 72°C before cooling to 10°C. PCR products were separated using 2% agarose gel electrophoresis and visualized with Et-Br staining. PCR reactions were performed in duplicates for each species and only reproducible fragments were scored.

### Statistical analysis

DNA fragments were scored as present (1)/absent (0) for each reaction and were assembled in a binary data matrix table. Genetic similarities and bootstrap analysis were performed using the FreeTree program [45]. Dendrograms were depicted with the TreeView software (http://taxonomy.zoology.gla.ac.uk/rod/treeview.html). An allele-based estimate was used to calculate mean heterozygosity over loci among all members of the population using GenAlEx 6.4 [46]. Genotypic variations were assessed across various populations by means of analysis of molecular variance (AMOVA) and the significance of the resulting variance components and inter-population genetic distances were tested using 999 random permutations.

A Bayesian model-based clustering approach for identification of the genetic structure in the edible herb germplasm was performed using STRUCTURE 2.3.4 [47]. The program was run using the admixture model with 10 independent replicate runs per K value (number of clusters) ranging from 1 to 10. Each run involved a burn-in period of 50000 iterations, and a post burn-in simulation length of 50000. Validation of the most likely number of clusters K was performed with the Structure Harvester (http://taylor0.biology.ucla.edu/structureHarvester).

## Abbreviations

AMOVA: Analysis of molecular variance
PCR: Polymerase chain reaction
